# Infection Dynamics and Host Responses to Two IPNV Isolates in Liver of Atlantic Salmon (*Salmo salar*)

**DOI:** 10.3390/pathogens14121245

**Published:** 2025-12-05

**Authors:** Valeria Aguilar Quiñones, Fabian Grammes, Victor Boyartchuk, Jacob Seilø Torgersen

**Affiliations:** 1 AquaGen AS, 7462 Trondheim, Norwayjacob.seilo.torgersen@aquagen.no (J.S.T.); 2 Centre for Integrative Genomics (CIGENE), Department of Animal and Aquacultural Sciences, Norwegian University of Life Sciences, 1432 Ås, Norway

**Keywords:** IPNV, emerging isolates, QTL-insensitive, viral dynamics, transcriptomics

## Abstract

The infectious pancreatic necrosis virus (IPNV) used to be one of the largest loss factors in Atlantic salmon farming. Since 2009, marker-assisted selection for resistance to IPN, targeting a single major quantitative trait locus (QTL), has led to a ten-fold decrease in the number of IPN outbreaks in Norway. However, some IPN-related problems remain, due to isolates of the virus which seem to bypass the resistance mechanism of the QTL. We comparatively characterized a classical isolate affected by the IPN-QTL (cIPNV) and an isolate that circumvents the QTL-based protection (rIPNV). Using both in vivo and in vitro challenges, the viral infection dynamics and host responses were evaluated by RT-qPCR and by gene ontology (GO) enrichment analysis from the RNA sequencing data of infected hepatocytes and the whole liver. Overall, cIPNV showed rapid replication with pronounced lytic cytopathology and enrichment for DNA damage, apoptosis and cell cycle disruption GO terms, while rIPNV exhibited slower accumulation of viral RNA and a transcriptional footprint consistent with pro-survival states in hepatocytes. While further research is needed to resolve the causality of QTL evasion, this work provides a first characterization of the pathogenicity of emerging QTL-insensitive IPNV isolates.

## 1. Introduction

Infectious pancreatic necrosis (IPN) is a viral disease mainly affecting salmonids, such as Atlantic salmon (*Salmo salar*) and rainbow trout (*Oncorhynchus mykiss*), at the start-feeding stage. A second susceptibility window for Atlantic salmon smolts occurs during the first weeks after sea transfer [1], probably triggered by the physiological changes that fish undergo during this period. Affected individuals exhibit a darker skin colour and a characteristic swimming behaviour, commonly described as corkscrew-like [2,3,4]. Post-mortem examination of the fish reveals further signs such as swollen abdomen, capillary engorgement, haemorrhagic puncta in pyloric caeca and pale liver [3,5]. Surviving fish from an IPN outbreak that are transferred to the sea appear to grow normally and be immune; therefore, mortality at this stage is very low [1]. However, a fraction of these fish become stunted, growing poorly and looking thinner and darker than the other fish [1,6].

The aetiological agent of this disease is IPN virus (IPNV) which enters the host fish primarily through the oral and gill routes, disseminating to the rest of the internal organs through the bloodstream [2]. In addition, epidermal wounds may act as a secondary entry route [2,7,8]. Viral transmission occurs mainly horizontally, but vertical infection routes to the eggs via the gonadal fluids could also occur, since the virus can adsorb to sperm cells and be found in the ovarian fluid [9], posing a transmission risk. The main target organs are the pancreas and liver, which develop severe necrosis of pancreatic acinar cells as well as focal or generalized necrosis in liver tissue, respectively [1].

IPNV belongs to the *Birnaviridae* viral family and *Aquabirnavirus* genus (species *Aquabirnavirus salmonidae*) [10]. It is a non-enveloped virus with an icosahedral capsid of around 60 nm in diameter [11] and a bi-segmented linear dsRNA genome of around 6 kb. Segment A encodes for the viral proteins VP2, VP3 and VP4, expressed as a polyprotein that further renders the individual proteins by the autoproteolytic activity of protease VP4. VP2 constitutes the major capsid component, and it is expressed as a premature protein (pVP2) that is later processed by proteases to result in the mature protein form and three additional peptides that will stay associated with the viral particle [12]. VP3 is located inside the virion interacting with the genomic segments forming a ribonucleoprotein (RNP) complex. In addition, VP3, as part of the RNP complex, captures pVP2 to initiate viral assembly and the formation of immature viral particles that will become infectious after the VP2 maturation process [13]. Additionally, segment A also codes for VP5, which is expressed by leaky scanning using a secondary open reading frame. The function of IPNV-VP5 is not fully clear yet, but it has been reported that the protein could support viral production through its antiapoptotic activity as well as being involved in the inhibition of the interferon (IFN) response [14,15,16]. The viral polymerase (VP1) is encoded by segment B and corresponds to an RNA-dependent RNA polymerase [11,17] which is found both freely inside the virion and attached to the genomic segments. The viral genome also has 5′- and 3′-UTR sequences at the ends of each segment. The 5′-UTR acts as an internal ribosomal entry site (IRES) commanding translation and it is also at these sites where VP1 is bound to (VPg) and serves as a protein-primer to initiate second-strand RNA synthesis, for which a specific stem-loop structure at the 3′-UTR is needed [18].

Knowing viral protein functionality is key to have a better understanding of the pathogenesis of the virus and potential host–pathogen interactions. Great effort has been made to obtain this knowledge, as described in the previous paragraphs, but still, we do not fully know the complete infectious cycle of IPNV. Nonetheless, similarities can be found by looking at another well-known birnavirus, such as infectious bursal disease virus (IBDV), which shares features such as genome type and organization, virion structure and symmetry or viral proteins [10]. IBD is a highly contagious and immunosuppressive viral disease in chickens, where two dominant epidemic strains circulate, the very virulent IBDV (vvIBDV) and the newly emerging novel variant IBDV (nVarIBDV) [19]. Unlike the lethal vvIBDV, the nonlethal nVarIBDV exhibits a more subtle pathogenicity, allowing it to partially evade the immune protection provided by existing vvIBDV vaccines. In poultry, IBDV mainly affects young chicks (3–6 weeks of age) and results in high losses. The virus can also affect older birds, but these are more resistant to clinical disease [20]. B-cells are the main target cells for IBDV and are rapidly destroyed by the virus, leading to immunosuppression. Here we see a parallelism with IPNV as, in that case, the virus is most severe to young salmonids and its main target cells (pancreatic acinar cells) are also destroyed fast. In addition, both viruses can establish acute or persistent infections, with survivors becoming carriers of the disease [21,22].

**Figure 1:**
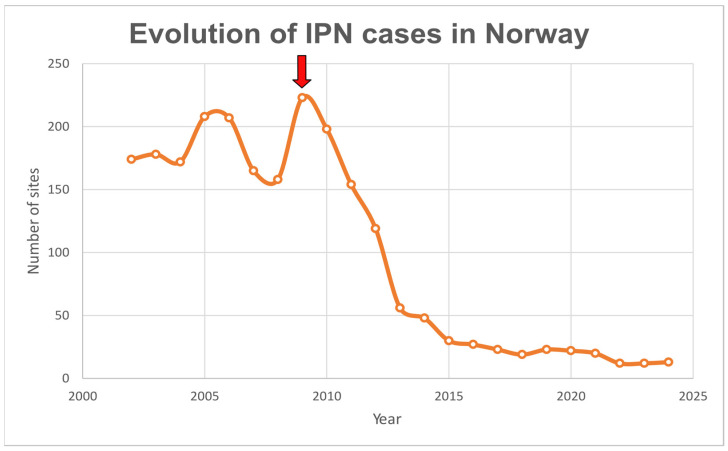
Number of sites with infectious pancreatic necrosis (IPN) cases in Norwegian farms during the period 2002–2024. Red arrow indicates the start of the implementation of selection based on the IPN-QTL in Atlantic salmon breeding programmes. Data retrieved from several Norwegian Fish Health reports (2009, 2014 and 2025 editions) [30,31,32].

IPN was listed as a notifiable disease by the World Organization for Animal Health (WOAH, formerly OIE) until 2005. The removal of the disease from the list was due to its global distribution and the lack of significant benefit from international reporting, since major salmon-farming countries were already affected. Therefore, it was concluded that IPN did not meet the necessary criteria for listing, since regular reporting would not improve disease management or conservation outcomes [23]. In addition, advancement in management practices and scientific research also contributed to the delisting. Such practices include early-stage vaccination and the implementation of surveillance programmes for early detection along with biosecurity measures at production sites [4,24]. From the year 2009, the use of selective breeding based on the IPN-QTL (described independently by Houston [25] and Moen [26]) has had the most significant impact, leading to a marked decline in IPN cases in Norway (Figure 1). In Norway, isolates from the Spjarup (Sp) strain (genogroup 5) are the most prevalent in Atlantic salmon farms. However, more recently, IPNV isolates to which the IPN-QTL does not appear to provide resistance to have emerged, not only in Norway, but also in Scotland and Chile [27,28,29].

In this paper, we describe differences in viral dynamics between two different IPNV isolates (V1244 and Vir410/2018). We also compare the host responses of the two isolates using transcriptomics, to further characterize the pathogenesis of the emerging IPNV isolates. The Vir410/2018 isolate corresponds to one of the emerging isolates that also affect QTL fish (also referred to in this paper as QTL-insensitive or recent isolate; rIPNV for short), while V1244 represents classical IPNV that is affected by the IPN-QTL (also referred to in this paper as QTL-sensitive or classical isolate; cIPNV for short).

## 2. Materials and Methods

### 2.1. Buffers and Cell Media

- Wash buffer: 1× Hank’s Balanced Salt Solution without magnesium and calcium (HBSS −Mg/−Ca), 1 mM EDTA, 10 mM HEPES (pH 7.4) stored at 4 °C.
- Collagenase buffer: 1× HBSS with magnesium and calcium (HBSS +Mg/+Ca), 10 mM HEPES, 150 U/mL collagenase (pH 7.5) stored at 4 °C.
- Hepatocyte culture media: Leibovitz’s L-15 Medium, GlutaMAX™ Supplement (L-15) supplemented with 10% fetal bovine serum (FBS), 1× penicillin-streptomycin (Pen-Strep), 2.5 µg/mL Amphotericin B, 2 mM ascorbic acid-2P and 2 mM nicotinamide.
- ASG-10 culture media: L-15 supplemented with 10% FBS and 1× Pen-Strep.
- Infection media: L-15 supplemented with 2% FBS and 1× Pen-Strep.

### 2.2. Viruses, Viral Propagation and Titration

Two different isolates of IPNV belonging to the Sp strain were used for the infection experiments (V1244—also known as NVI-015, and Vir410/2018—also known as H15Y12no1Sea23Y12) and were kindly provided by Prof. Espen Rimstad (NMBU, Norway). Atlantic salmon gill cells 10 (ASG-10), obtained from the Norwegian Veterinary Institute, were used to propagate the IPNV isolates at a multiplicity of infection (MOI) of 0.01. In short, ASG-10 cells were grown in T75 flasks at 20 °C and maintained as described elsewhere [33]. When reaching ~80% confluency, the viral inoculum was prepared, adding the appropriate amount of virus to the required volume of infection media and transferred to the cell monolayer. Flasks were monitored for cytopathic effect (CPE) and supernatant was collected when ~90% of cells had detached. Clarification of the collected supernatants was performed by centrifugation at 2800× *g* for 10 min at 4 °C and aliquots were stored at −80 °C until used.

For viral titration, tissue culture infectious dose 50 (TCID_50_) assays were performed. Briefly, ASG-10 cells were cultured in flat-bottomed 96-well plates on the day prior to infection. After 7 days post-infection (dpi), cells were fixed and stained with 1% crystal violet solution containing 4% formaldehyde and the viral titre was calculated following the Reed–Muench method [34].

### 2.3. Phylogenetic Analysis

Genomic sequences from segment A of different IPNV isolates (Table S1) were retrieved, including the ones used in this study and the emerging isolates in Norway, Scotland and Chile [27,28,29]. These sequences represent the six different genogroups established by Blake et al. [35] (erratum numbering system) and cluster within the viral species *Aquabirnavirus salmonidae*. The VP2 protein sequences were translated using Geneious Prime software (version 2024.0.3) to be used in the phylogenetic analysis. In total, 29 full-length and 4 partial VP2 sequences were used. Multiple alignment of the protein sequences was performed with Clustal omega 1.2.2 and used as input to build the phylogenetic tree in Geneious Prime following the neighbour-joining method. The robustness of the resulting tree was evaluated through bootstrap analysis with 1000 replicates. Annotation and visualization of the phylogenetic tree was achieved using the Interactive Tree of Life (iTOL; version 6) tools [36].

### 2.4. Comparative Sequence and Structural Analysis of Viral Proteins

Genomic sequences from segment A of rIPNV (GenBank ID: MH562009) and cIPNV (GenBank ID: AY379740) isolates were obtained and translated into the different viral proteins using Geneious Prime software (version 2024.0.3). In addition, the VP2 protein sequences from the Gx (isolate Chicken/UK/UK661/1989) and Gt (isolate Chicken/Cuba/Soroa/1998) IBDV strains (UniProtKB accession: Q82635 and UniProtKB accession: Q9WI42, respectively) were also retrieved to be aligned. Protein sequence alignments of all viral proteins obtained were made with Clustal omega 1.2.2 pairwise.

Structural analysis of VP2 proteins from IPNV and IBDV was performed using PyMol software (version 3.1.4.1). For IPNV, visualizations were made based on the IPNV virion structure (PDB ID: 9GG2), whose VP2 protein sequence is the same as the one from the QTL-insensitive isolate. The cIPNV VP2 version was made by using the Mutagenesis Wizard from PyMol and changing the corresponding residues. For IBDV, the structures used corresponded to the Gx strain virion (PDB ID: 7VRP) and the Gt strain virion (PDB ID: 7VRN). The electrostatic potential surface of the proteins was calculated by applying the APBS Electrostatics plug-in available in PyMol software.

### 2.5. Investigating IPNV Isolates In Vivo and In Vitro

#### 2.5.1. In Vivo Challenge Test in Atlantic Salmon Fry

Two in vivo challenge tests were performed in parallel at VESO Aqualab (Namsos, Norway), referred to from now on as tank or bucket challenge tests, respectively. The tank challenge test consisted of two different groups of Atlantic salmon fry (AquaGen AS families) ready for start-feeding that were infected with either Vir410/2018 (rIPNV) or V1244 (cIPNV) following VESO Aqualab’s immersion challenge protocol (husbandry details during the trials can be found in Table S2). Injuries and deformities were part of the exclusion criteria of the study. The initial population of the tank infected with cIPNV was 2000 fish, while there were 1965 fish in the rIPNV-infected tank. It was conducted in single tanks with a large number of fish per tank to maximize statistical power and ensure that potential differences in mortality and disease progression could be detected. A similar single-tank challenge performed in 2021 at the same facility yielded comparable results, supporting the reproducibility of the current findings (Figure S1 and Table S3). For the bucket challenge, 124 fry were challenged with rIPNV, following the same protocol. The fish population used for the tank challenge test had equitable phenotype frequencies based on the IPN-QTL genotypes, which were also balanced for the bucket challenge population (Tables S4 and S5). Fish from the bucket challenge were sampled at 15 and 23 days post-challenge (dpc), and stored in RNALater solution for downstream RNA extraction.

#### 2.5.2. In Vitro Challenge Test in Hepatocyte Cultures

To establish a primary hepatocyte culture, 200 g fish from the Fish Centre facility at NMBU were euthanized by a sharp blow to the head, and the hepatocytes were isolated as previously described [37]. Briefly, the liver was perfused with wash buffer for 5 min to remove the blood and subsequently with collagenase buffer for another 5 min. The liver was dissected and placed in a Petri dish to be cut into smaller pieces. Collagenase buffer was poured along with the liver pieces into an autoclaved flask containing a sterile magnet and incubated for 1 h at 15 °C at low spinning speed. After incubation, cells were collected using a 70 µm cell strainer and centrifuged at 100× *g* for 5 min at 4 °C. Supernatant was removed and the cells were resuspended in 5 mL HBSS −Mg/−Ca. Centrifugation and resuspension steps were repeated once before counting the cells using a hemocytometer. Cells were then seeded accordingly in plates previously coated with hyaluronic acid solution (1 mg/mL).

Liver cells were cultured at 20 °C in hepatocyte culture media for a period of two days. After that time, the cells were washed with PBS and fresh culture media was added, supplemented additionally with growth factors (5 ng/mL epidermal growth factor (EGF) and 3 ng/mL hepatocyte growth factor (HGF)). At day 7 post-culture, the media was replaced once again to hepatocyte culture media without growth factors.

Two independent challenge tests using primary hepatocytes from four different fish (F1–F4) were performed. Challenge 1 consisted of cells from F1 while the hepatocytes from the remaining fish (F2–F4) were part of challenge 2. Hepatocytes from the different fish were cultured separately in six-well plates and were infected in duplicates at MOI 0.1 with cIPNV or rIPNV for 1 h at 15 °C. After incubation time, the viral inoculum was removed and replaced with infection media also containing Amphotericin B (2.5 µg/mL). The cells were kept at 15 °C and monitored daily for CPE. Both cells and supernatants were collected for RNA extraction at 4 and 7 dpi for challenge 1 and at 2 and 4 dpi for challenge 2. Microscopy pictures were taken at 4 and 7 dpi using the EVOS™ XL Core Imaging System (Invitrogen™, Waltham, MA, USA) with the EVOS™ 4X achromat objective (0.13 NA/10.58 WD) (ThermoFisher Scientific, Waltham, MA, USA).

### 2.6. DNA Extraction and Genotyping

DNA extraction and genotyping was performed on samples from both challenge tests. DNA was extracted from cultured hepatocytes from challenge 2 (in vitro challenge test) using the DNeasy Blood & Tissue kit (Qiagen^®^, Hilden, Germany) in accordance with the manufacturer’s spin-column protocol for DNA purification from animal cells. The same kit was used for the control fish samples from the bucket challenge test but following the purification protocol for tissues using a rotor-stator homogenizer (TissueRuptor^®^ II, Qiagen^®^). For the remaining fry fish that were part of the challenge tests, a fin-clip was collected into 2D matrix tubes prefilled with lysis buffer. DNA was extracted by a crude extraction method i.e., lysis of the tissue with Proteinase K (Qiagen^®^). Quality control of a subset of samples was performed by gel electrophoresis prior to genotyping.

After DNA extraction, the samples were genotyped using Applied Biosystems™ Axiom™ Genotyping Solution (Microarray Analysis, ThermoFisher Scientific, Waltham, MA, USA), formerly known as Affymetrix (AFFY). The genotyping service was performed either at the laboratories Blue Analytics (Blue Analytics AS, Bergen, Norway) or CIGENE (NMBU, Ås, Norway), following the manufacturer’s instructions. The DNA samples were amplified and fragmented prior to being added to the customized axiom array (SNP-chip) for Atlantic salmon. The custom genotyping array was designed and fabricated by Life Technologies (ThermoFisher Scientific) and its affiliates based on a set of target sequences and other information provided by AquaGen AS, called “Ssa70Kv3”. The raw data was processed and quality checked before genotype calling was performed using Axiom Power Tools (APT 2.12.0) and further analysis was performed to assess the genotypes.

### 2.7. Gene Expression

#### 2.7.1. RNA Extraction from Diverse Sources

RNA was extracted from tissue, cells and/or supernatants using different spin-column technology extraction kits. For tissue samples collected from the in vivo challenge test, total RNA was extracted from liver stored in RNALater and from the remaining internal organs using RNeasy Mini kit (Qiagen^®^). A rotor-stator homogenizer (TissueRuptor^®^ II, Qiagen^®^) was used to disrupt and homogenize the tissue. The same kit, but following the corresponding manufacturer’s protocol for extraction from cells, was used for the hepatocyte cell samples from the in vitro challenge test. Finally, viral RNA was extracted using PureLink™ Viral RNA/DNA Mini Kit (Invitrogen™) from clarified supernatants following the producer’s protocol with minor adjustments. To prepare the lysate, 200 µL of clarified supernatant were mixed with 25 µL proteinase K (20 mg/mL) and 200 µL of Lysis Buffer containing carrier RNA (5.6 µg per sample), vortexed and incubated at 56 °C for 15 min. Next, absolute ethanol was added to the samples to precipitate the RNA. These were vortexed and incubated at room temperature for 5 min before transferring to the spin columns. After the recommended centrifugation steps for binding and washing, RNA was eluted in RNase-free water previously heated at 90 °C. The RNA samples were labelled and stored at −80 °C until processed downstream.

#### 2.7.2. Determination of Viral Load and Viral Production by RT-qPCR

Two-step RT-qPCR was conducted to determine the viral load on RNA samples (cells and supernatant) from the challenge tests. For this, the RNA was extracted as previously described and converted to cDNA using the SuperScript™ IV First-Strand cDNA Synthesis System kit (ThermoFisher Scientific). Briefly, for the RNA samples coming from either tissue (internal organs) or cells (primary hepatocytes), 1500 ng RNA and 200 ng RNA, respectively, were used as input for the cDNA synthesis reaction. To assess viral load in the supernatant samples from the in vitro challenge tests, 5 µL RNA was used as the reaction input. The template RNA was annealed to gene-specific reverse primers (2 µM primer against IPNV-VP3 and 0.5 µM primer against *ef1a*) by heating the RNA–primer mix at 42 °C for 5 min followed by 1 min on ice. For the supernatant samples, annealing was only made with the 2 µM gene-specific reverse primer against IPNV-VP3. The reverse transcription mix was made following the manufacturer’s protocol, in which the mixtures were incubated at 50 °C for 10 min to initialize reverse transcription. The reaction was inactivated by incubating the mixtures at 80 °C for 10 min. At this moment, the cDNA was either used directly in the qPCR setup or stored at −20 °C until used.

To run qPCR from the cDNA samples from cells or tissue, primer pairs for amplifying *ef1a* (GenBank ID: 100136525) and IPNV-VP3 (10 µM primer mix) were used, whereas only primers for IPNV-VP3 were used for the supernatant cDNA samples. The qPCR reactions were set up in triplicates (including non-template controls for each primer pair) using PowerUp™ SYBR™ Green Master Mix 2X (Applied Biosystems™, Waltham, MA, USA) in accordance with the kit’s protocol and run using the CFX96 Touch Real-Time PCR Detection System (Bio-rad Laboratories, Inc., Hercules, CA, USA). The cDNA input for qPCR, corresponding to the in vivo challenge samples, was 15 ng cDNA. A total of 2 ng cDNA were utilized for the cell samples from the in vitro challenge test and 2 µL cDNA was the input for the supernatant samples. All primer sequences used for both cDNA and qPCR reactions, as well as the thermocycling conditions, can be found in Tables S6 and S7, respectively.

Relative viral RNA load was calculated for the in vitro challenge test cell samples at each timepoint for comparison between isolates. Total fold-change (FC), as well as the log_2_ (FC), were calculated and defined as ΔCt values of cIPNV relative to the ΔCt values of rIPNV. The ΔCt values were obtained by subtracting the Ct values of the *ef1a* (housekeeping gene) assay from the Ct values of the IPNV qPCR test obtained with isolates cIPNV or rIPNV.

#### 2.7.3. RNA Sequencing

Total RNA samples from both in vivo and in vitro challenge tests were analyzed by RNA sequencing (Table 1). The RNA sources used were liver (in vivo challenge test) and hepatocytes (in vitro challenge test). RNA concentration and quality were assessed before sending the samples for sequencing using NanoDrop Eight (ThermoFisher Scientific) and TapeStation 4150 (Agilent Technologies, Santa Clara, CA, USA). The samples that fulfilled the sample requirements for RNA sequencing given by the service provider were sent in for the library preparation. The in vivo challenge test RNA samples and those from the in vitro challenge 1 were sequenced by Novogene (Novogene Company Limited, Cambridge, UK), while the sequencing service for samples from the in vitro challenge 2 was performed by BMKGENE (Biomarker Technologies, Cheshire, UK). Despite having used two different service providers, the sequencing technology and data output was the same. The sequencing platform used was Illumina NovaSeq X (PE150 strategy), generating a data output of 20 million read pairs per sample.

RNA sequencing files (.fastq) were processed using a custom snakemake script [38] using the following steps: (i) Raw paired-end (PE) reads were quality controlled using FastQC (version 0.12.0) [39]; (ii) The raw PE reads were subsequently quality trimmed using cutadapt (version 4.1) [40], removing traces of the Illumina 3′ and 5′ adapter, trimming low-quality bases from the end of the reads (parameter-q 25) and only keeping reads with a minimum length; (iii) Trimmed PE reads were again quality checked using FastQC; and (iv) Trimmed PE reads were aligned to the Atlantic salmon genome (Ssal_v3.1: GCA_905237065) using STAR (v2.7.9a) [41]. The genome index included ensemble 106 gene annotations. Reads per gene were counted using the parameter: -quantMode GeneCounts.

Bucket challenge: Total library sizes were normalized to account for bias in sample composition, using the trimmed mean of m-values approach. Differential gene expression analysis was performed using the limma/voom [42] pipeline in R (version 4.4.2). Principal component analysis (PCA) revealed no clear influence of either infection status (individuals with qPCR-confirmed IPNV infection), infection timepoint, or IPN-QTL genotypes (Figure S2); thus, differential expression was estimated for Control vs. rIPNV-infected fish.

In vitro challenges: Reads from technical replicates were pooled, after confirming that they were indeed identical via PCA. Total library sizes were normalized to account for bias in sample composition, using the trimmed mean of m-values approach. Differential gene expression analysis was performed using the limma/voom [42] pipeline in R (version 4.4.2) using the individual from which the cells originated as a blocking factor and the infection (control, rIPNV- or cIPNV-infected) as an experimental factor.

### 2.8. Gene Ontology and Pathway Enrichment Analysis

Enrichment analyses and comparative assessments of the transcriptome datasets were conducted using g:Profiler (2016 version) [43] and the Enrichment Map application in Cytoscape (version 3.10.3) [44,45]. Lists of significantly differentially expressed genes (DEGs; *p* < 0.05, log_2_FC ≥ 1.0) were converted to human gene symbols using the Ensembl ortholog database via BioMart (https://www.ensembl.org/; accessed on 4 August 2025). For genes with multiple entries, log_2_FC values were summed to enable single-gene input compatibility in Cytoscape. Gene Ontology (GO) enrichment analyses were performed in g:Profiler using the human genome as the background reference, applying the Benjamini–Hochberg false discovery rate (FDR) correction with a significance threshold of *p* < 0.05. The resulting GO terms were subsequently visualized and compared using Enrichment Map. The plots were made using generative AI (ChatGPT-5 model, OpenAI).

### 2.9. Statistical Tests

To test whether mortality differed by genotype within the two different groups during the tank challenge test, 3 × 2 contingency tables were built separately for cIPNV- and rIPNV-infected tanks (genotype [QQ, Qq, qq] × outcome [dead, alive]). Tests were run in R (version 4.3.3) using Pearson’s chi-square test (α = 0.05). If the overall 3 × 2 test was significant, post hoc pairwise genotype comparisons were conducted separately in 2 × 2 chi-square tests. The resulting *p*-values from the post hoc tests were adjusted for multiple testing using the Bonferroni method.

## 3. Results

### 3.1. Phylogenetic Analysis of IPNV Isolates

**Figure 2:**
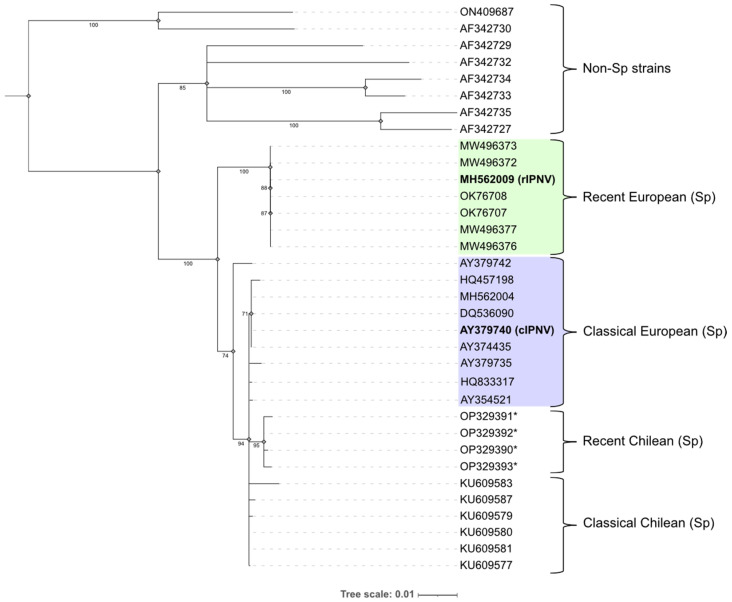
Phylogenetic tree built using the neighbour-joining method and rooted at midpoint showing the relationship between the VP2 proteins from different IPN virus (IPNV) isolates represented by their NCBI accession number. Bootstrap values are indicated at the corresponding branches and the tree scale units correspond to substitutions per amino acid residue. The isolates belonging to genogroups 1–4 and 6 are annotated as “Non-Sp strains”. Genogroup 5 isolates (Sp) are further classified into different subgroups contrasting recent and classical isolates, as well as their origin (Europe or Chile). The recent European isolates are coloured in green, while the classical European are coloured in purple. The isolates used in this study (rIPNV and cIPNV) are highlighted in bold. Asterisks (*) denote partial VP2 sequences.

To investigate the relationship between the two IPNV isolates used in this study, a phylogenetic tree was constructed based on the VP2 amino acid sequences (Figure 2). The analysis confirmed that both isolates belonged to the Sp strain (genogroup 5). The recently emerged isolates in Norway and Scotland formed their own cluster within the Sp strain clade, suggesting all these isolates (including rIPNV) are closely related. Within the remaining Sp strain sequences there is a mix between Chilean and European isolates with the Chilean variants that caused an outbreak in genetically resistant fish during 2022 [29] forming their own subgroup. A second subgroup consisted of some of the classical European isolates, including cIPNV.

### 3.2. Sequence Analysis of Vir410/2018 and V1244 Shows Main Differences Reside in the VP2 Protein

The viral protein sequences from segment A of both isolates were analyzed and compared to have an overview of the mutations that each of the isolates carry. The protein alignments (Figure S3) showed that the main differences between the isolates resided in the VP2 (Table 2) and VP5 proteins. The QTL-insensitive isolate exhibits 12 different mutations in VP2 compared to the classical isolate, most of which are located within the hypervariable region of the protein. The majority of these mutations are considered to be radical substitutions [46] (e.g., E248R) with only a few of them being conservative (e.g., V278A), based on the physicochemical properties of the amino acids. Mutations on positions 217, 221 and 247 have been linked to being virulence motifs in previous studies [47,48,49,50]. The other main difference was found in the VP5 protein, which had an early stop codon at position 92, rendering a VP5 protein 14 amino acids shorter for the rIPNV. Besides the premature stop codon, there were two other amino acid substitutions at positions 36 and 45 (leucine to proline in both cases). Finally, for VP4 and VP3 proteins there were minor changes, with one and two mutations found, respectively (D77G; Q22K and R68H).

**Figure 3:**
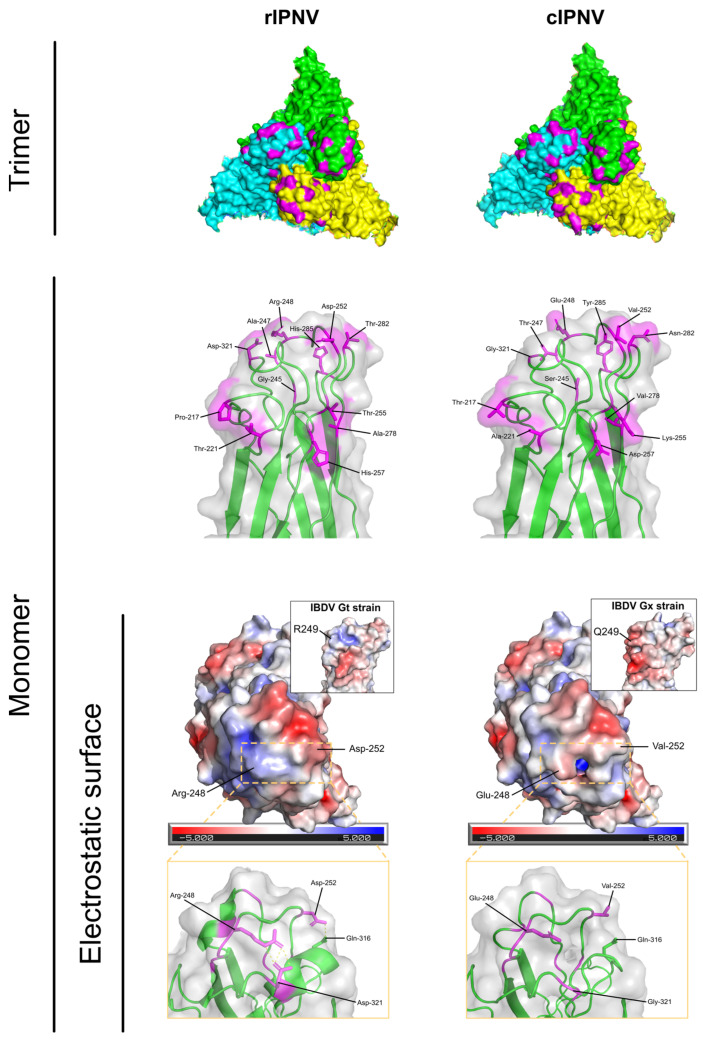
Structural comparison of the VP2 protein from the recent (rIPNV) and classical (cIPNV) isolates. VP2 trimers are shown in the first row where monomers are coloured in green, yellow and cyan. The mutated residues are highlighted in magenta. VP2 monomers in closer detail are shown next, with the labelled mutated amino acids in magenta, displaying their side chains. Lastly, the electrostatic potential surface of the VP2 monomers is shown from a top view. Labelled within the dashed box are amino acid substitutions that had a big impact in the electrostatic surface (E248R and V252D). A zoomed-in lateral view of the dashed box region is represented in the last row, showing the gain/loss of polar contacts between the amino acids involved. Embedded in the electrostatic surface representations are visualizations of the VP2 protein from two different IBDV strains (Gt and Gx), exhibiting a similar shift in the electrostatic surface in this region (from negative to positive potential). All representations were made using PyMol v.3.1.4.1.

To have a better understanding of how the 12 amino acid substitutions could impact VP2 from a structural and functional point of view, visualizations of the protein in 3D conformation (Figure 3) were made based on the IPNV virion model (PDB ID: 9GG2). The mutated residues were localized at the top of the spike of the VP2 protein, with many of them being exposed to the surface. Therefore, it is very likely that they are involved in intermolecular interactions and that mutations in these positions could influence binding with potential interactors (viral receptor, host proteins or other host factors). The mutations in some of these positions are of low impact, since the amino acid shift is slightly synonymous (e.g., V278A) or implies minor changes in polarity or protein flexibility (e.g., A221T and S245G). However, there are other mutations that have a higher impact on the physicochemical properties of the protein (E248R, V252D, D257H and G321D) that affect its electrostatic surface (Figure 3) or that rigidify the backbone (T217P). Such alterations could also influence the polar contacts within the protein, as shown in Figure 3.

A similar mutation to E248R (IBDV Q249R) was also observed between a very virulent IBDV strain and its attenuated form (Gx and Gt, respectively), that likewise resulted in a change from a negative to positive electrostatic potential patch in that region (Figure 3). In addition, a proline was also introduced in the Gt strain (A222P), similar to that observed in rIPNV (T217P), that impacts the protein structurally, since proline residues are α-helix breakers and introduce turns in the protein structure. The total number of mutations between the VP2 of both IBDV strains resulting in amino acid substitutions is 12 (Figure S3).

These results taken together suggest that the changes within the VP2 protein of IPNV potentially impact virulence (similarly to the observed in IBDV strains) and molecular interactions that could affect cell tropism or immune evasion.

### 3.3. The Isolates Display Differences in Viral Dynamics Based on In Vivo and In Vitro Challenge Tests

**Figure 4:**
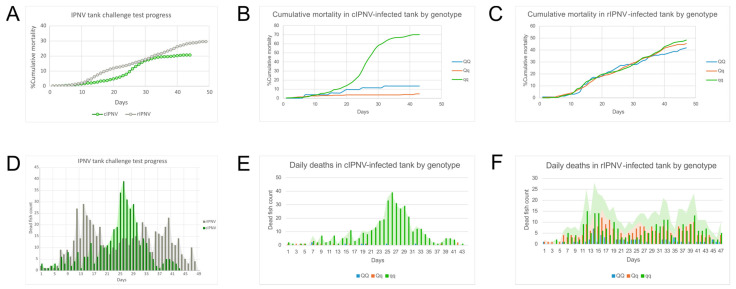
Tank challenge tests’ progress. (**A**) Cumulative mortality during the tank challenge test for the QTL-sensitive isolate (green) and the QTL-insensitive isolate (grey). (**B**,**C**) Cumulative mortality by genotype (QQ, Qq or qq) for cIPNV- and rIPNV-infected tanks, respectively. (**D**) Death counts per day during the tank challenge test for cIPNV (green) and rIPNV (grey). (**E**,**F**) Daily deaths by genotype for cIPNV- and rIPNV-infected tanks, respectively.

A tank challenge test in Atlantic salmon fry was performed to evaluate how the two different IPNV isolates behave when infecting the host and to assess differences in the infection progress. Cumulative mortality for the V1244 (cIPNV) isolate reached 20.7%, while it was 29.6% for the Vir410/2018 (rIPNV) isolate (Figure 4A). The difference in sensitivity to the QTL for each isolate becomes evident when the cumulative mortality is subdivided by genotype, revealing the higher mortality due to cIPNV in the qq group and the equal distribution of mortality due to rIPNV across all host genotypes (Figure 4B,C). Looking at the deaths per day, we observed that mortality peaked earlier in the rIPNV-infected tank (14 dpc) and happened to be more spread out over time with a second mortality peak by 40 dpc, whereas, for the cIPNV-infected group, there was a single mortality peak between days 21 and 33 in which the majority of the deaths occurred (Figure 4D–F), suggesting that the isolates behave differently. In parallel to the tank challenge test, a smaller challenge was run with fry infected with the QTL-insensitive isolate, from which samples were taken to assess viral load and perform a transcriptomic analysis. The cumulative mortality was also registered and resulted in 17.4% mortality during a 23-day period (Figure S4), which follows the same trend observed in the tank challenge test. Comparing the infection progress for both isolates, it seems that the classical isolate progresses following an acute infection characterized by the presence of a mortality peak, while we observe a more continuous infection progress trend for the rIPNV.

**Figure 5:**
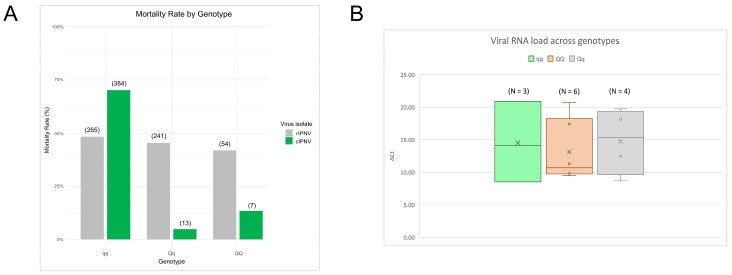
IPN-QTL effect upon mortality and viral RNA load across genotypes. (**A**) Mortality rate for each genotype in the tank challenge test for cIPNV (44 dpc) represented in green, and rIPNV (47 dpc) represented in grey. The numbers at the top in brackets indicate the number of dead fish within each group. (**B**) rIPNV viral RNA load by genotype from bucket challenge internal organ samples (23 days post-challenge) expressed as ΔCt values. A total of 7 samples per genotype were assessed, showing in the plot those for which rIPNV was detected by qPCR (N).

To verify that the recent isolate was insensitive to the IPN-QTL, the mortality rate was calculated for each genotype group (QQ, Qq and qq) and chi-square tests were made to analyze if differences between the groups were significant. The frequencies of dead and alive fish by genotype during the tank challenge test for both viral isolates can be found in Tables S8 and S9 in the Supplementary Materials. Mortality rates for the known QTL-sensitive isolate differed across genotypes, showing a clear inclination towards the qq fish, in contrast to the heterozygote and QQ fish (Figure 5A), the differences being significant (Tables S10 and S11). On the other hand, for the recent isolate, we do not observe this pattern in the mortality rates, and these are evenly distributed across all genotypes, indicating that the isolate is not affected by the QTL. Furthermore, the isolate was detected by qPCR in fish from the bucket challenge, irrespective of their genotype (Figure 5B).

**Figure 6:**
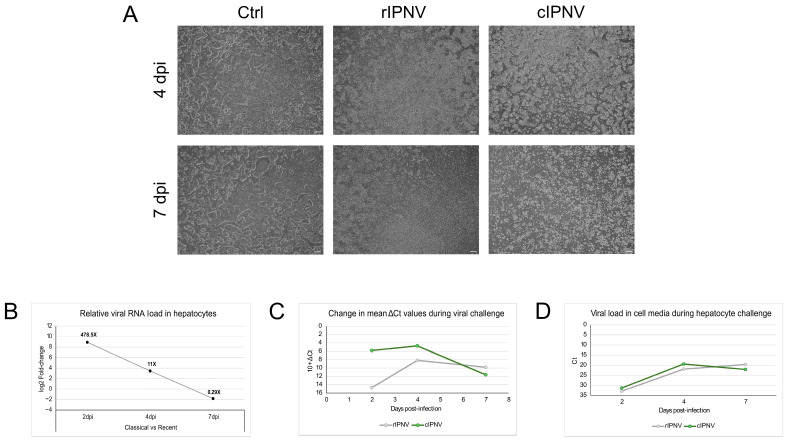
Infection progress and viral load measurements during hepatocyte challenge in vitro. (**A**) Microscopy pictures of hepatocytes during the challenge with rIPNV and cIPNV taken at 4 and 7 days post-infection (dpi). Pictures from non-infected hepatocytes (Ctrl) are shown in the first column. Changes in cell density and morphology can be observed between the groups for the different timepoints. The white scale bar at the bottom right corner of each picture represents 200 µm. (**B**) Relative viral RNA load in hepatocytes during the in vitro challenge test contrasting the classical and recent isolates expressed as log_2_ fold-change. The numbers at the top of the datapoints show the fold-change values. (**C**) Change in the mean ΔCt values in hepatocytes during the viral challenge for the recent (grey) and classical (green) isolates. An arbitrary unit of 10 was added systematically to all ΔCt values to shift the dataset above zero for visualization purposes. This transformation does not alter the relationships among data points, as all analyses were conducted on the original data. (**D**) Viral load in the cell supernatant for the recent (grey) and classical (green) isolates during the hepatocyte challenge expressed as Ct values. A similar plot was obtained when the qPCR Ct values obtained for the cell culture supernatant were normalized to the sample’s total RNA yield.

An in vitro challenge test in primary hepatocytes derived from QQ fish was also conducted. CPE was monitored throughout the infection period (Figure 6A), and viral RNA load was quantified both within the cells and in the cell media at different timepoints. At 4 dpi, hepatocytes infected with either rIPNV or cIPNV showed a rounder and contracted appearance, deviating from the characteristic cobblestone-like morphology of healthy hepatocytes. These morphological changes were more pronounced in the hepatocyte cultures infected with the classical isolate. By 7 dpi, differences between the isolates became more evident. The rIPNV-infected wells exhibited minor CPE patches and a cell density similar to the uninfected controls, despite obvious signs of compromised cell health. Conversely, extensive destruction of the cell monolayer was observed in the cIPNV-infected wells, with a significant reduction in cell density due to virus-induced lysis. Altogether, these observations suggest that the two isolates also display different viral dynamics in vitro, with the cIPNV inducing CPE more rapidly and extensively.

Results from the qPCR assays (Figure 6B–D) also support the latter. At early stages of infection, we find higher intracellular levels of viral RNA in hepatocytes infected with cIPNV compared to those infected with rIPNV. This difference is particularly pronounced at 2 dpi, where RNA levels for cIPNV are 478.5 times higher, suggesting a fast and abundant viral replication. However, at the later stage (7 dpi), the trend reverses, with higher levels of rIPNV RNA detected inside the cells. A similar pattern was seen in the viral load measurements of the cell media. On days 2 and 4 post-infection, there are slightly more cIPNV particles in the supernatant (Ct values 31.3 and 19.4, respectively) compared to the recent isolate (Ct values 32.9 and 21.9, respectively). By 7 dpi, however, rIPNV is more abundant in the cell media. The drop in the Ct values between days 2 and 4 is indicative of active viral replication and virions being released to the media. The decline in viral load for the classical isolate after day 4 could be due to extensive cell death, suggesting a classical acute infection dynamic reaching the peak, in this case at 4 dpi. In contrast, we do not observe this pattern for the recent isolate. Instead, it appears to follow a more persistent or continuous infection dynamic characterized by lower viral levels early on that gradually increase and are maintained over time.

### 3.4. Enrichment Analysis of Host Responses to IPNV

We analyzed RNA sequencing data from the infection challenges, both in vivo (bucket challenge) and in vitro (hepatocyte challenge) separately. For the in vivo and in vitro challenge, we found 27,759 and 29,288 genes to be expressed (CPM > 1), respectively. PCA did not show separation of the different timepoints in the challenges; hence, differential expression analysis was based on contrasting non-infected control against the viral isolates. Within the expressed genes in liver samples, 3081 were differentially expressed (1082 upregulated and 1999 downregulated). In the hepatocyte cultures, there were 1069 genes differentially expressed for the rIPNV-infected group (369 upregulated and 700 downregulated) and 1118 for the cIPNV-infected hepatocytes (463 upregulated and 655 downregulated). Overall, we found a higher number of DEGs in the liver compared to the primary hepatocyte cultures, with a greater fraction of the genes exhibiting downregulation in all three datasets.

**Figure 7:**
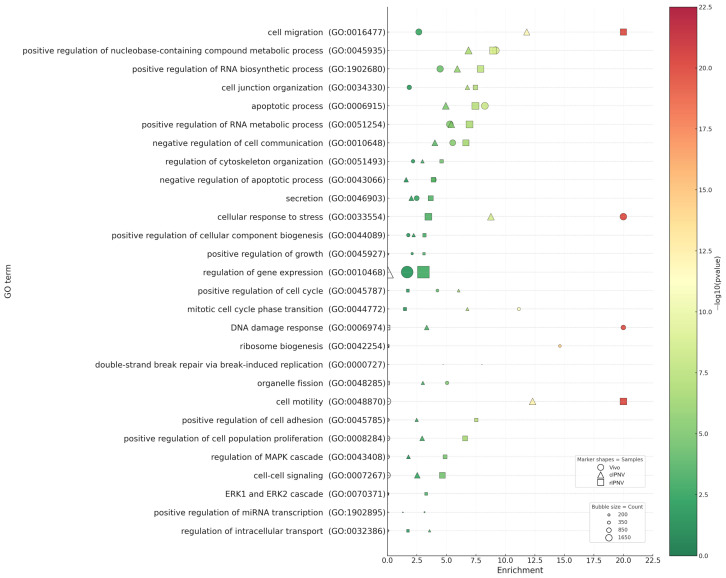
Gene ontology (GO) enrichment plot of GO terms linked to cellular processes for each dataset (Vivo, cIPNV and rIPNV) and its significance (colour-coded). The non-filled markers shown are indicative of the absence of the GO term in the corresponding dataset.

**Figure 8:**
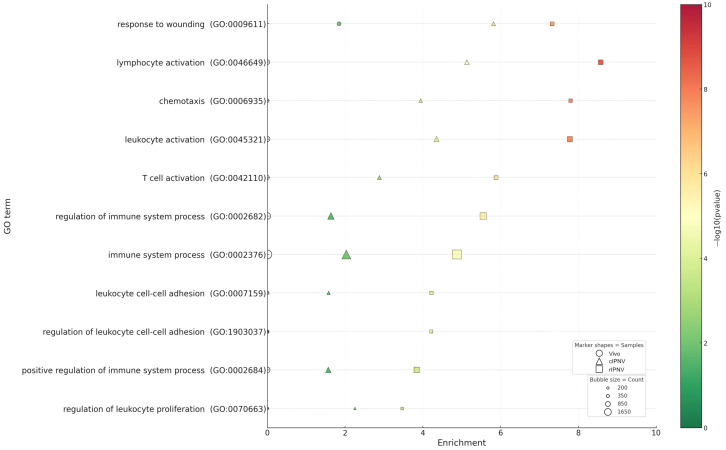
GO enrichment plot of immune-related GO terms for each dataset (Vivo, cIPNV and rIPNV) and its significance (colour-coded). The non-filled markers shown are indicative of the absence of the GO term in the corresponding dataset.

Irrespective of the IPNV isolate, GO analysis across datasets (Figure 7) revealed markedly stronger transcriptional responses in the liver of infected fish (*p*-values up to 10^−34^) compared with cultured primary hepatocytes infected with either cIPNV or rIPNV (*p*-values up to 10^−13^ and 10^−23^ respectively). The most significantly enriched GO categories in the in vivo dataset (*p* = 10^−5^ to 10^−34^) encompassed RNA metabolism, responses to stress and DNA damage, as well as cell cycle regulation. Within the hepatocyte responses, we observed that many of the GO categories were shared between the viruses, although the significance of most of them was higher for the QTL-insensitive isolate. The top GO terms for rIPNV included processes like cell migration and motility, RNA metabolism, cell proliferation and anti-apoptosis (*p* = 10^−4^ to 10^−23^). For cIPNV, categories related to RNA metabolism or cell motility and migration were also among the top ones shared with rIPNV, but also some others related to stress response, cell cycle regulation, apoptosis and DNA damage were observed high in the significant list (*p* = 10^−4^ to 10^−13^). Common GO terms shared between the in vivo dataset and both IPNV isolates from in vitro-challenged hepatocytes included categories such as cellular response to stress (GO:0033554), apoptotic process (GO:0006915) and several related to RNA metabolism (GO:0045935, GO:1902680 and GO:0051254). Notably, immune-related GO terms were only significantly present in the in vitro datasets (Figure 8), besides response to wounding (GO:0009611), which was present across all three. The significance of such GO terms was also higher for rIPNV compared to cIPNV with *p*-values ranging from 10^−4^ to 10^−9^ and from 10^−2^ to 10^−6^, respectively.

## 4. Discussion

We acknowledge that the use of a single-tank design for the in vivo challenge tests limits the ability to account for potential tank-level variation, which is an important consideration in experimental aquaculture studies. However, the large number of individuals included in this study give sufficient statistical power. Moreover, the comparable results obtained in a previous challenge conducted in 2021 at the same facility (Figure S1) suggest that the observed trends are robust and reproducible. The comparative studies conducted between the two IPNV isolates in this paper reveal that they have different strategies to establish infection, reflected by their divergent viral dynamics and host responses. Based on the challenge tests performed, the cIPNV isolate establishes an acute infection marked by a rapid mortality peak and extensive cell death in vivo and in vitro, respectively. On the other hand, the rIPNV isolate seems to drive a more continuous and persistent infection based on the absence of a clear, single infection peak characteristic of acute infections during the in vivo challenge and higher cell survival in vitro. These observations suggest that these two IPNV isolates employ different infection strategies. The QTL-sensitive isolate overwhelms the host defences quickly in genetically susceptible fish, while the QTL-insensitive isolate evades clearance and maintains a low-grade infection across the entire fish population.

The 12 mutations on the VP2 protein could help the recent isolate to evade the host defences, since they are located in the region of the protein involved in antigenicity [51] and, as shown in Figure 3, some of these changes have a significant impact in the electrostatic surface, likely implying alterations in intermolecular interactions with host factors. In addition, the more subtle pathogenicity observed during rIPNV infections compared to cIPNV could also contribute to rIPNV-like isolates emerging from the viral pool and causing disease. Such isolates are able to overcome the biological resistance provided by the IPN-QTL, similar to what has been observed in IBDV between vvIBDV and nVarIBDV isolates [19], in which the low pathogenic isolate (nVarIBDV) evades the protection given by vvIBDV-based vaccines.

Not only might the mutations in the VP2 protein explain the differences in pathogenicity, but the ones in VP5 could also have a contributing effect. As mentioned earlier in the paper, the role of VP5 in IPNV remains to be elucidated, although previous research indicates that it could have an antiapoptotic function or inhibit IFN production [14,15,16]. VP5 is a non-structural protein of IPNV and may tentatively serve to modulate virulence, as many other known viral non-structural proteins do. It is a common strategy for viruses to have some of their non-structural proteins serve as effectors to evade the host defences and enhance virulence by antagonizing IFN production [52,53] or shutting down cellular protein synthesis to favour production of the viral components [54,55], among other strategies. The function of VP5 in IBDV is better understood and it could be that IPNV-VP5 has a similar role to its avian counterpart. IBDV-VP5 modulates viral egression and has been shown to associate to the plasma membrane where it accumulates [56]. Such accumulation is toxic to the cell and alters its permeability, facilitating viral release and inducing cell lysis. Given this, and hypothesizing that the IPNV-VP5 could follow the same mechanism, the mutations found in rIPNV-VP5 could impact such function and make the virus less lytic, in line with the observed during the in vitro challenge assay in hepatocytes.

Thus, mutations within proteins VP2 and/or VP5 might be the key to explain the difference in the behaviour of both viruses, how the host responds to them and the reason why the QTL-based protection might fail for the rIPNV isolate. During the in vivo challenge tests, infection with the recent isolate was observed in individuals regardless of their genotype (Figure 5), further supported by qPCR detection of the virus in 13 out of 21 samples from the bucket challenge. However, with a sample size of *n* = 7 per genotype, we had limited power to estimate genotype-specific differences in viral RNA load and, therefore, firm conclusions cannot be drawn. A larger sample size would be required to estimate any genotypic effects more precisely in this regard. Nonetheless, the mortality rate per genotype calculated from the tank challenge test for rIPNV validates that all fish are susceptible to the recent isolate, irrespective of their genotype status.

In addition to examining how the variation in the viral proteins might have an impact upon infection, we also evaluated how the host responds. Across the three RNA sequencing datasets, enrichment analyses revealed a stronger and broader transcriptional response in the challenged fish than in cultured primary hepatocytes. This is to some extent expected, since bulk liver RNA captures not only hepatocytes, but the integrated response of multiple cell types that are part of the organ, such as sinusoidal endothelial cells or biliary epithelial cells, among others. Furthermore, there are additional systemic cues contributing as well that are not present in the in vitro model using cultured hepatocytes, yielding larger effect sizes and strongly enriched GO terms. Within the top enriched GO categories, we found pathways related to RNA metabolism, cellular response to stress and cell death, suggesting that IPNV infection interferes with host RNA machinery and processing as well as apoptosis. Both processes are well known to be hijacked by viral pathogens and have also been reported as modulated across diverse viruses and host species [57,58,59,60].

Previous work has sought to characterize gene expression profiles during IPNV infection in live salmonids using different approaches [61,62,63,64,65,66], but to our knowledge this is the first RNA sequencing study in cultured hepatocytes from Atlantic salmon in response to two IPNV isolates that differ in their sensitivity to the IPN-QTL. Within the in vitro setting, infection with the QTL-insensitive isolate resulted in a larger number of significantly enriched GO terms compared to the QTL-sensitive isolate. This could be explained by the differences in infection dynamics between the isolates. In the cIPNV-infected hepatocytes, where qPCR data and microscopy images indicate more advanced cytopathic effects (Figure 6), it is likely that a large proportion of the cells were already dead or undergoing apoptosis when sampled. Consequently, the major transcriptional response may have already occurred earlier in the infection, leading to the limited differential expression observed. In contrast, rIPNV infection appears to progress slower, enabling it to capture the host transcriptional response still active. The enriched GO terms in rIPNV-infected hepatocytes provide insights into the nature of these responses. Processes such as the positive regulation of cell population proliferation (GO:0008284), the negative regulation of the apoptotic process (GO:0043066) and the positive regulation of growth (GO:0045927) suggest that hepatocytes may be activating compensatory survival mechanisms to counteract viral stress and preserve cellular function.

Remarkably, GO enrichment of immune-related categories was significant in vitro (particularly for rIPNV), but was not significantly enriched in the liver (Figure 8). This could be due to sampling and timing, as immune transcriptional peaks in vivo may occur earlier or in non-parenchymal areas and, therefore, may be diluted in bulk liver RNA dominated by the parenchymal transcriptome, since liver leukocyte content is relatively low [67]. In addition, the lack of enrichment for canonical interferon or cytokine pathways suggests that IPNV acts stealthily in the liver before (or without) eliciting a robust liver-wide immune response. This data supports an apparently stealthy hepatic phenotype of IPNV rather than excluding immune activation in other cells or tissues. Immune modulation by IPNV has been reported previously in primary immune organs like the head kidney or spleen [65,68], as well as in immune organ-derived cell lines such as SHK-1 [68], using qPCR analysis.

We would like to stress that around 30% of the functional gene annotations of Atlantic salmon genes present in the DEG lists are lost during the enrichment analysis pipeline, as the annotations the Enrichment Map application from Cytoscape uses are based on human gene symbols. Therefore, Atlantic salmon genes that lack an equivalent human gene symbol are removed from downstream analyses, which could affect the interpretation of the results. On top of that, there is also a large number of Atlantic salmon genes whose annotation is itself missing or incomplete.

This work provides a first step towards characterizing the pathogenicity of a QTL-insensitive IPNV isolate. Overall, our results point to two contrasting infection strategies: a rapidly lytic cIPNV versus a slower, more subtle rIPNV with different host transcriptional footprints. The underlying mechanisms linking VP2/VP5 variation to these phenotypes and the means by which rIPNV circumvents QTL-based protection remain unclear and warrant further investigation.

## Figures and Tables

**Table 1:** Overview of RNA samples for each challenge test used for the differential expression analysis.

Study	N Samples	Sequencing Provider
Bucket challenge (in vivo)	41	NovoGene
In vitro challenge 1	14	NovoGene
In vitro challenge 2	34	BMKGene

**Table 2:** Amino acid substitutions in the VP2 protein from cIPNV and rIPNV. The colour scale is indicative of the electrostatic potential (blue: positive; white: neutral; red: negative).

Position	cIPNV	rIPNV	
217	T	P	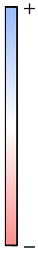
221	A	T	
245	S	G	
247	T	A	
248	E	R	
252	V	D	
255	K	T	
257	D	H	
278	V	A	
282	N	T	
285	Y	H	
321	G	D	

## Data Availability

The RNA sequencing datasets used in this study are publicly available in the European Nucleotide Archive (ENA) with accession numbers E-MTAB-16378 (in vivo challenge dataset) and E-MTAB-16379 (in vitro challenge dataset).

## Supplementary Materials

The following supporting information can be downloaded at https://www.mdpi.com/article/10.3390/pathogens14121245/s1, Figure S1: IPNV tank challenge test progress from 2021; Figure S2: PCA plots from the RNA sequencing analysis (bucket challenge); Figure S3: Protein alignments from viral proteins; Figure S4: Cumulative mortality during bucket challenge; Table S1: List of IPNV isolate sequences used in the construction of the phylogenetic tree; Table S2: Husbandry details during the tank and bucket challenge tests; Table S3: IPN-QTL genotype frequencies from IPNV-challenged fry in the tank challenge test from 2021; Tables S4 and S5: IPN-QTL genotype frequencies from IPNV-challenged fry in the tank and bucket challenge tests, respectively; Table S6: Primer sequences used for cDNA synthesis and qPCR reactions; Table S7: Thermocycling conditions used for qPCR; Tables S8 and S9: Frequencies of dead and alive fish by genotype during the tank challenge test for the cIPNV- and rIPNV-infected groups, respectively; Table S10: Mortality by genotype in the IPNV tank challenge: Pearson’s chi-square tests using 3 × 2 contingency tables; Table S11: Mortality by genotype in the IPNV tank challenge: post-hoc pairwise chi-square tests (Bonferroni corrected) for both viral isolates.

## Abbreviations

The following abbreviations are used in this manuscript:

IPNV	Infectious Pancreatic Necrosis Virus
IPN	Infectious Pancreatic Necrosis
QTL	Quantitative Trait Locus
cIPNV	Classical Infectious Pancreatic Necrosis Virus
rIPNV	Recent Infectious Pancreatic Necrosis Virus
GO	Gene Ontology
pVP2	Premature VP2
RNP	Ribonucleoprotein
IFN	Interferon
IRES	Internal Ribosomal Entry Site
UTR	Untranslated Region
VPg	Genomic-bound VP1
IBDV	Infectious Bursal Disease Virus
IBD	Infectious Bursal Disease
vvIBDV	Very Virulent Infectious Bursal Disease Virus
nVarIBDV	Novel Variant Infectious Bursal Disease Virus
WOAH	World Organization for Animal Health
Sp	Spjarup
HBSS	Hank’s Balanced Salt Solution
L-15	Leibovitz’s L-15 Medium, GlutaMAX™ Supplement
FBS	Fetal Bovine Serum
ASG-10	Atlantic Salmon Gill Cells 10
MOI	Multiplicity of Infection
CPE	Cytopathic Effect
TCID_50_	Tissue Culture Infectious Dose 50
dpi	Days Post-infection
EGF	Epidermal Growth Factor
HGF	Hepatocyte Growth Factor
FC	Fold-change
PE	Paired-end
PCA	Principal Component Analysis
DEG	Differential Expressed Gene
FDR	False Discovery Rate
CPM	Counts per Million

## References

[1] Roberts R.J., Pearson M.D. (2005). Infectious Pancreatic Necrosis in Atlantic Salmon, *Salmo salar* L.. J. Fish Dis..

[2] Wolf K. (1988). Fish Viruses and Fish Viral Diseases.

[3] Roberts R.J. (2012). Fish Pathology.

[4] Munro E.S., Midtlyng P.J. (2011). Infectious Pancreatic Necrosis and Associated Aquatic Birnaviruses. Fish Diseases and Disorders. Volume 3: Viral, Bacterial and Fungal Infections.

[5] Frasca S., Wolf J.C., Kinsel M.J., Camus A.C., Lombardini E.D., Terio K.A., McAloose D., Leger J.S. (2018). Chapter 39—Osteichthyes. Pathology of Wildlife and Zoo Animals.

[6] Bruno D.W., Poppe T.T., Noguera Patricia A. (1996). A Colour Atlas of Salmonid Diseases.

[7] Munang’andu H.M., Fredriksen B.N., Mutoloki S., Dalmo R.A., Evensen Ø. (2013). Antigen Dose and Humoral Immune Response Correspond with Protection for Inactivated Infectious Pancreatic Necrosis Virus Vaccines in Atlantic Salmon (*Salmo salar* L). Vet. Res..

[8] Munang’andu H.M., Fredriksen B.N., Mutoloki S., Brudeseth B., Kuo T.-Y., Marjara I.S., Dalmo R.A., Evensen Ø. (2012). Comparison of Vaccine Efficacy for Different Antigen Delivery Systems for Infectious Pancreatic Necrosis Virus Vaccines in Atlantic Salmon (*Salmo salar* L.) in a Cohabitation Challenge Model. Vaccine.

[9] Smail D.A., Munro E.S. (2008). Isolation and Quantification of Infectious Pancreatic Necrosis Virus from Ovarian and Seminal Fluids of Atlantic Salmon, *Salmo salar* L.. J. Fish Dis..

[10] Delmas B., Attoui H., Ghosh S., Malik Y.S., Mundt E., Vakharia V.N., Ictv Report Consortium (2019). ICTV Virus Taxonomy Profile: Birnaviridae. J. Gen. Virol..

[11] Dobos P., Hill B.J., Hallett R., Kells D.T., Becht H., Teninges D. (1979). Biophysical and Biochemical Characterization of Five Animal Viruses with Bisegmented Double-Stranded RNA Genomes. J. Virol..

[12] Galloux M., Chevalier C., Henry C., Huet J.-C., Costa B.D., Delmas B. (2004). Peptides Resulting from the pVP2 C-Terminal Processing Are Present in Infectious Pancreatic Necrosis Virus Particles. J. Gen. Virol..

[13] Pedersen T., Skjesol A., Jørgensen J.B. (2007). VP3, a Structural Protein of Infectious Pancreatic Necrosis Virus, Interacts with RNA-Dependent RNA Polymerase VP1 and with Double-Stranded RNA. J. Virol..

[14] Hong J.-R., Gong H.-Y., Wu J.-L. (2002). IPNV VP5, a Novel Anti-Apoptosis Gene of the Bcl-2 Family, Regulates Mcl-1 and Viral Protein Expression. Virology.

[15] Santi N., Sandtrø A., Sindre H., Song H., Hong J.-R., Thu B., Wu J.-L., Vakharia V.N., Evensen Ø. (2005). Infectious Pancreatic Necrosis Virus Induces Apoptosis in Vitro and in Vivo Independent of VP5 Expression. Virology.

[16] Skjesol A., Aamo T., Hegseth M.N., Robertsen B., Jørgensen J.B. (2009). The Interplay between Infectious Pancreatic Necrosis Virus (IPNV) and the IFN System: IFN Signaling Is Inhibited by IPNV Infection. Virus Res..

[17] Dobos P. (1995). The Molecular Biology of Infectious Pancreatic Necrosis Virus (IPNV). Annu. Rev. Fish Dis..

[18] Rivas-Aravena A., Muñoz P., Jorquera P., Diaz A., Reinoso C., González-Catrilelbún S., Sandino A.M. (2017). Study of RNA-A Initiation Translation of The Infectious Pancreatic Necrosis Virus. Virus Res..

[19] Jiang N., Wang G., Zhang W., Wang Y., Niu X., Huang M., Gao L., Li K., Cui H., Liu C. (2023). A Single Mutation of VP2 Is Responsible for the Lethality and Antigenicity Differences between Novel Variant and Very Virulent IBDV Strains. Transbound. Emerg. Dis..

[20] Dey S., Pathak D.C., Ramamurthy N., Maity H.K., Chellappa M.M. (2019). Infectious Bursal Disease Virus in Chickens: Prevalence, Impact, and Management Strategies. Vet. Med..

[21] Abdul R., Murgia M.V., Rodriguez-Palacios A., Lee C.-W., Saif Y.M. (2013). Persistence and Tissue Distribution of Infectious Bursal Disease Virus in Experimentally Infected SPF and Commercial Broiler Chickens. Avian Dis..

[22] Julin K., Johansen L.-H., Sommer A.-I., Jørgensen J.B. (2015). Persistent Infections with Infectious Pancreatic Necrosis Virus (IPNV) of Different Virulence in Atlantic Salmon, *Salmo salar* L.. J. Fish Dis..

[23] OIE Aquatic Animal Health Standards Commission (2005). Report of the Meeting of the OIE Aquatic Animal Health Standards Commission.

[24] Sommerset I., Wiik-Nielsen J., Moldal T., Oliveira V.H.S., Svendsen J.C., Haukaas A., Brun E. (2024). Norwegian Fish Health Report 2023.

[25] Houston R.D., Haley C.S., Hamilton A., Guy D.R., Tinch A.E., Taggart J.B., McAndrew B.J., Bishop S.C. (2008). Major Quantitative Trait Loci Affect Resistance to Infectious Pancreatic Necrosis in Atlantic Salmon (*Salmo salar*). Genetics.

[26] Moen T., Baranski M., Sonesson A.K., Kjøglum S. (2009). Confirmation and Fine-Mapping of a Major QTL for Resistance to Infectious Pancreatic Necrosis in Atlantic Salmon (*Salmo salar*): Population-Level Associations between Markers and Trait. BMC Genom..

[27] Hillestad B., Johannessen S., Melingen G.O., Moghadam H.K. (2021). Identification of a New Infectious Pancreatic Necrosis Virus (IPNV) Variant in Atlantic Salmon (*Salmo salar* L.) That Can Cause High Mortality Even in Genetically Resistant Fish. Front. Genet..

[28] Benkaroun J., Muir K.F., Allshire R., Tamer C., Weidmann M. (2021). Isolation of a New Infectious Pancreatic Necrosis Virus (IPNV) Variant from a Fish Farm in Scotland. Viruses.

[29] Godoy M., Kibenge M.J.T., Montes de Oca M., Pontigo J.P., Coca Y., Caro D., Kusch K., Suarez R., Burbulis I., Kibenge F.S.B. (2022). Isolation of a New Infectious Pancreatic Necrosis Virus (IPNV) Variant from Genetically Resistant Farmed Atlantic Salmon (*Salmo salar*) during 2021–2022. Pathogens.

[30] Moldal T., Wiik-Nielsen J., Oliveira V.H.S., Svendsen J.C., Sommerset I. (2025). Norwegian Fish Health Report 2024.

[31] Hjeltnes B. (2014). Fish Health Report 2013.

[32] Johansen R., Kongtorp R.T., Bornø G., Ringkjøb Skjelstad H., Olsen A.B., Flesjå K., Colquhoun D., Ørpetveit I., Hansen H., Garseth Å.H. (2009). The Health Situation in Farmed Salmonids 2008.

[33] Gjessing M.C., Aamelfot M., Batts W.N., Benestad S.L., Dale O.B., Thoen E., Weli S.C., Winton J.R. (2018). Development and Characterization of Two Cell Lines from Gills of Atlantic Salmon. PLoS ONE.

[34] Reed L.J., Muench H. (1938). A Simple Method of Estimating Fifty Per Cent Endpoints. Am. J. Epidemiol..

[35] Blake S., Ma J.-Y., Caporale D.A., Jairath S., Nicholson B.L. (2001). Phylogenetic Relationships of Aquatic Birnaviruses Based on Deduced Amino Acid Sequences of Genome Segment A cDNA. Dis. Aquat. Org..

[36] Letunic I., Bork P. (2024). Interactive Tree of Life (iTOL) v6: Recent Updates to the Phylogenetic Tree Display and Annotation Tool. Nucleic Acids Res..

[37] Datsomor A.K., Wilberg R., Torgersen J.S., Sandve S.R., Harvey T.N. (2023). Efficient Transfection of Atlantic Salmon Primary Hepatocyte Cells for Functional Assays and Gene Editing. G3 Genes Genomes Genet..

[38] Mölder F., Jablonski K.P., Letcher B., Hall M.B., Tomkins-Tinch C.H., Sochat V., Forster J., Lee S., Twardziok S.O., Kanitz A. (2021). Sustainable Data Analysis with Snakemake. F1000Research.

[39] Simon A. (2010). FastQC.

[40] Martin M. (2011). Cutadapt Removes Adapter Sequences from High-Throughput Sequencing Reads. EMBnet. J..

[41] Dobin A., Davis C.A., Schlesinger F., Drenkow J., Zaleski C., Jha S., Batut P., Chaisson M., Gingeras T.R. (2013). STAR: Ultrafast Universal RNA-Seq Aligner. Bioinformatics.

[42] Law C.W., Chen Y., Shi W., Smyth G.K. (2014). Voom: Precision Weights Unlock Linear Model Analysis Tools for RNA-Seq Read Counts. Genome Biol..

[43] Kolberg L., Raudvere U., Kuzmin I., Adler P., Vilo J., Peterson H. (2023). G:Profiler—Interoperable Web Service for Functional Enrichment Analysis and Gene Identifier Mapping (2023 Update). Nucleic Acids Res.

[44] Shannon P., Markiel A., Ozier O., Baliga N.S., Wang J.T., Ramage D., Amin N., Schwikowski B., Ideker T. (2003). Cytoscape: A Software Environment for Integrated Models of Biomolecular Interaction Networks. Genome Res..

[45] Merico D., Isserlin R., Stueker O., Emili A., Bader G.D. (2010). Enrichment Map: A Network-Based Method for Gene-Set Enrichment Visualization and Interpretation. PLoS ONE.

[46] Zhang J. (2000). Rates of Conservative and Radical Nonsynonymous Nucleotide Substitutions in Mammalian Nuclear Genes. J. Mol. Evol..

[47] Santi N., Vakharia V.N., Evensen Ø. (2004). Identification of Putative Motifs Involved in the Virulence of Infectious Pancreatic Necrosis Virus. Virology.

[48] Shivappa R.B., Song H., Yao K., Aas-Eng A., Evensen Ø., Vakharia V.N. (2004). Molecular Characterization of Sp Serotype Strains of Infectious Pancreatic Necrosis Virus Exhibiting Differences in Virulence. Dis. Aquat. Org..

[49] Rodríguez Saint-Jean S., de las Heras A.I., Pérez Prieto S.I. (2010). The Persistence of Infectious Pancreatic Necrosis Virus and Its Influence on the Early Immune Response. Vet. Immunol. Immunopathol..

[50] Mutoloki S., Jøssund T.B., Ritchie G., Munang’andu H.M., Evensen Ø. (2016). Infectious Pancreatic Necrosis Virus Causing Clinical and Subclinical Infections in Atlantic Salmon Have Different Genetic Fingerprints. Front. Microbiol..

[51] Coulibaly F., Chevalier C., Delmas B., Rey F.A. (2010). Crystal Structure of an Aquabirnavirus Particle: Insights into Antigenic Diversity and Virulence Determinism. J. Virol..

[52] Klaitong P., Smith D.R. (2021). Roles of Non-Structural Protein 4A in Flavivirus Infection. Viruses.

[53] Wang T., Wei F., Jiang Z., Song J., Li C., Liu J. (2022). Influenza Virus NS1 Interacts with 14-3-3ε to Antagonize the Production of RIG-I-Mediated Type I Interferons. Virology.

[54] Smiley J.R. (2004). Herpes Simplex Virus Virion Host Shutoff Protein: Immune Evasion Mediated by a Viral RNase?. J. Virol..

[55] Jagger B.W., Wise H.M., Kash J.C., Walters K.-A., Wills N.M., Xiao Y.-L., Dunfee R.L., Schwartzman L.M., Ozinsky A., Bell G.L. (2012). An Overlapping Protein-Coding Region in Influenza A Virus Segment 3 Modulates the Host Response. Science.

[56] Lombardo E., Maraver A., Espinosa I., Fernández-Arias A., Rodriguez J.F. (2000). VP5, the Nonstructural Polypeptide of Infectious Bursal Disease Virus, Accumulates within the Host Plasma Membrane and Induces Cell Lysis. Virology.

[57] Friedel C.C., Whisnant A.W., Djakovic L., Rutkowski A.J., Friedl M.-S., Kluge M., Williamson J.C., Sai S., Vidal R.O., Sauer S. (2021). Dissecting Herpes Simplex Virus 1-Induced Host Shutoff at the RNA Level. J. Virol..

[58] Bercovich-Kinori A., Tai J., Gelbart I.A., Shitrit A., Ben-Moshe S., Drori Y., Itzkovitz S., Mandelboim M., Stern-Ginossar N. (2016). A Systematic View on Influenza Induced Host Shutoff. eLife.

[59] Gervais O., Peñaloza C., Gratacap R., Papadopoulou A., Beltrán M., Henderson N.C., Houston R.D., Hassan M.A., Robledo D. (2023). Understanding Host Response to Infectious Salmon Anaemia Virus in an Atlantic Salmon Cell Line Using Single-Cell RNA Sequencing. BMC Genom..

[60] Yang Z., Luo W., Huang Z., Guo M., He X., Fan Z., Wang Q., Qin Q., Yang M., Lee X. (2022). Genome-Wide Analysis of Differentially Expressed mRNAs and lncRNAs in Koi Carp Infected with Koi Herpesvirus. Viruses.

[61] Woldemariam N.T., Agafonov O., Sindre H., Høyheim B., Houston R.D., Robledo D., Bron J.E., Andreassen R. (2020). miRNAs Predicted to Regulate Host Anti-Viral Gene Pathways in IPNV-Challenged Atlantic Salmon Fry Are Affected by Viral Load, and Associated with the Major IPN Resistance QTL Genotypes in Late Infection. Front. Immunol..

[62] Aedo J.E., Aravena-Canales D., Dettleff P., Fuentes-Valenzuela M., Zuloaga R., Rivas-Aravena A., Molina A., Valdés J.A. (2022). RNA-Seq Analysis Reveals the Dynamic Regulation of Proteasomal and Autophagic Degradation Systems of Rainbow Trout (Oncorhynchus Mykiss) Skeletal Muscle Challenged with Infectious Pancreatic Necrosis Virus (IPNV). Aquaculture.

[63] Tapia D., Kuznar J., Farlora R., Yáñez J.M. (2022). Differential Transcriptomic Response of Rainbow Trout to Infection with Two Strains of IPNV. Viruses.

[64] Lockhart K., McBeath A.J.A., Collet B., Snow M., Ellis A.E. (2007). Expression of Mx mRNA Following Infection with IPNV Is Greater in IPN-Susceptible Atlantic Salmon Post-Smolts than in IPN-Resistant Atlantic Salmon Parr. Fish Shellfish Immunol..

[65] McBeath A.J.A., Snow M., Secombes C.J., Ellis A.E., Collet B. (2007). Expression Kinetics of Interferon and Interferon-Induced Genes in Atlantic Salmon (*Salmo salar*) Following Infection with Infectious Pancreatic Necrosis Virus and Infectious Salmon Anaemia Virus. Fish Shellfish Immunol..

[66] Robledo D., Taggart J.B., Ireland J.H., McAndrew B.J., Starkey W.G., Haley C.S., Hamilton A., Guy D.R., Mota-Velasco J.C., Gheyas A.A. (2016). Gene Expression Comparison of Resistant and Susceptible Atlantic Salmon Fry Challenged with Infectious Pancreatic Necrosis Virus Reveals a Marked Contrast in Immune Response. BMC Genom..

[67] Taylor R.S., Ruiz Daniels R., Dobie R., Naseer S., Clark T.C., Henderson N.C., Boudinot P., Martin S.A.M., Macqueen D.J. (2022). Single Cell Transcriptomics of Atlantic Salmon (*Salmo salar* L.) Liver Reveals Cellular Heterogeneity and Immunological Responses to Challenge by *Aeromonas salmonicida*. Front. Immunol..

[68] Reyes-Cerpa S., Reyes-López F., Toro-Ascuy D., Montero R., Maisey K., Acuña-Castillo C., Sunyer J.O., Parra D., Sandino A.M., Imarai M. (2014). Induction of Anti-Inflammatory Cytokine Expression by IPNV in Persistent Infection. Fish Shellfish Immunol..

