# Supplementary material for: Infection Dynamics and Host Responses to Two IPNV Isolates in Liver of Atlantic Salmon (Salmo salar)
Source: Pathogens. 2025 Dec 5;14(12):1245. doi: 10.3390/pathogens14121245 (PMC12735744; doi:10.3390/pathogens14121245)
Supplement: Supplementary file 1 [file pathogens-14-01245-s001.zip › pathogens-3962685-supplementary.pdf]

Supplementary Materials

Table S1. List of IPNV isolate sequences used in the construction of the phylogenetic tree.

Accession number (NCBI)	Genogroup	Location
AF342727	1	United States
AF342735	1	United States
AF342729	2	United States
AF342732	3	Canada
AF342733	4	Canada
AF342734	4	Canada
AF342730	6	United States
ON409687	6	Sweden
AY354521	5	Norway
AY374435	5	Norway
AY379735	5	Norway
AY379740[a]	5	Norway
AY379742	5	Norway
DQ536090	5	Norway
HQ457198	5	Norway
HQ833317	5	Norway
KU609577	5	Chile
KU609579	5	Chile
KU609580	5	Chile
KU609581	5	Chile
KU609583	5	Chile
MH562004	5	Norway
MH562009[b]	5	Norway
MW496372	5	Norway
MW496373	5	Norway
MW496376	5	Norway
MW496377	5	Norway
OK076707	5	Scotland
OK076708	5	Scotland
OP329390*	5	Chile
OP329391*	5	Chile
OP329392*	5	Chile
OP329393*	5	Chile

* Partial VP2 sequence.

[a]cIPNV

[b]rIPNV

Table S2. Husbandry details during the tank and bucket challenge tests.

**Start date**	26.01.2022 (both tank and bucket challenges)
**End date**	11.03.2022 (tank) and 18.02.2022 (bucket)
**Fish average weight**	0.2 g
**Salinity**	Freshwater
**Stocking density**	Max 40 kg/m3
**Water temperature**	12 °C ± 1°C
**Water flow**	Adjusted according to minimum 70% oxygen in effluent water (over the sieve)
**Water discharge**	Tube overflow system
**Cleaning**	Once a day
**Photoperiod regime**	L:D = 24:0
**Feeding**	Automatic feeder
**Tank size**	0.6 m tank and 10 L bucket

Table S3. IPN-QTL genotype frequencies from IPNV-challenged fry in the tank challenge test from 2021.

**Host phenotype**	**Genotype**	**N**	**f (%)**
Resistant	QQ	1631	90.01
	Qq	171	9.43
Susceptible	qq	10	0.56
Unknown	Missing	0	0.00
**Total**		1812	100

Table S4. IPN-QTL genotype frequencies from IPNV-challenged fry in the tank challenge test.

**Host phenotype**	**Genotype**	**N**	**f (%)**
Resistant	QQ	181	8.7
	Qq	800	38.4
Susceptible	qq	1095	52.6
Unknown	Missing	5	0.2
**Total**		2194	100

Table S5. IPN-QTL genotype frequencies from IPNV-challenged fry in the bucket challenge test.

**Host phenotype**	**Genotype**	**N**	**f (%)**
Resistant	QQ	46	37
	Qq	36	29
Susceptible	qq	35	28
Unknown	Missing	7	6
**Total**		124	100

Table S6. Primer sequences used for cDNA synthesis and qPCR reactions.

Primer	Sequence (5′ → 3′)	Target	Purpose
Ef1a-qPCR-Fwd	CAATGGAGGCTGGCACTTTC	*ef1a*	qPCR
Ef1a-qPCR-Rv	GAACTTGCAGGCGATGTGAG	*ef1a*	qPCR
Ef1a-cDNA-Rv	CCGACGGTCGATCT	*ef1a*	cDNA synthesis
IPNV-qPCR-Fwd	AGAAGCCCAAAGGACCAGAC	IPNV-VP3	qPCR
IPNV-qPCR-Rv	GTACTTGAACTGCCCGGGA	IPNV-VP3	qPCR
IPNV-cDNA-Rv	TGGTTCTGGTTCTCGT	IPNV-VP3	cDNA synthesis

Table S7. Thermocycling conditions used for qPCR.

Step	Temperature (°C)	Time	Cycles
Uracil-DNA Glycosylase Activation	50	2 min	1
DNA polymerase Activation	95	2 min	1
Denaturation	95	15 s	
Annealing	59.5	15 s	40
Extension	72	1 min	
Denaturation	95	15 s	1
Melting Curve	60 → 95<br>(0.2 increment)	15 s	1

Table S8. Frequencies of dead and alive fry by genotype during the tank challenge for the cIPNV-infected group.

Status	Genotype	N	f (%)
Dead	QQ	7	1.7
	Qq	13	3.2
	qq	384	93.9
	Missing	5	1.2
**Total**		409	100
Alive	QQ	45	9.7
	Qq	256	55.2
	qq	163	35.1
	Missing	0	0.0
**Total**		464	100

Table S9. Frequencies of dead and alive fry by genotype during the tank challenge for the rIPNV-infected group.

Status	Genotype	N	f (%)
Dead	QQ	54	9.64
	Qq	241	43.04
	qq	265	47.32
	Missing	0	0.00
**Total**		560	100
Alive	QQ	75	11.6
	Qq	290	44.8
	qq	283	43.7
	Missing	0	0.0
**Total**		648	100

Table S10. Mortality by genotype in the IPNV tank challenge: Pearson's chi-square tests using 3 × 2 contingency tables.

Group	χ^2 statistic	df	p-value
rIPNV	2.13	2	0.3443
cIPNV	334.02	2	$< 2.2 \times 10^{-16}$

df: degrees of freedom

Table S11. Mortality by genotype in the IPNV tank challenge: post-hoc pairwise chi-square tests (Bonferroni corrected).

Tank	Contrast	p-value	Adjusted p-value (Bonferroni)
rIPNV-infected	QQ vs Qq	0.5328	1
	QQ vs qq	0.2179	0.6537
	Qq vs qq	0.3592	1
cIPNV-infected	QQ vs Qq	0.04103	0.1231
	QQ vs qq	< 0.000001	< 0.000001
	Qq vs qq	< 0.000001	< 0.000001

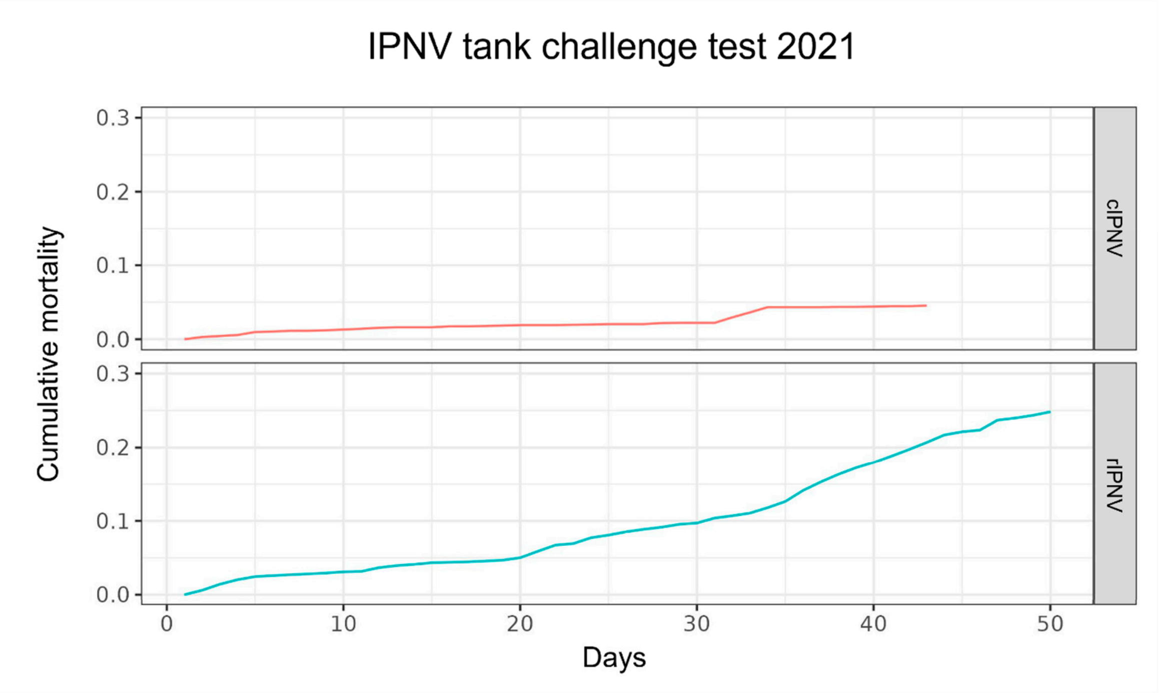

Figure S1. IPNV tank challenge test progress from 2021. Cumulative mortalities are represented for cIPNV- (top) and rIPNV-infected groups (bottom).

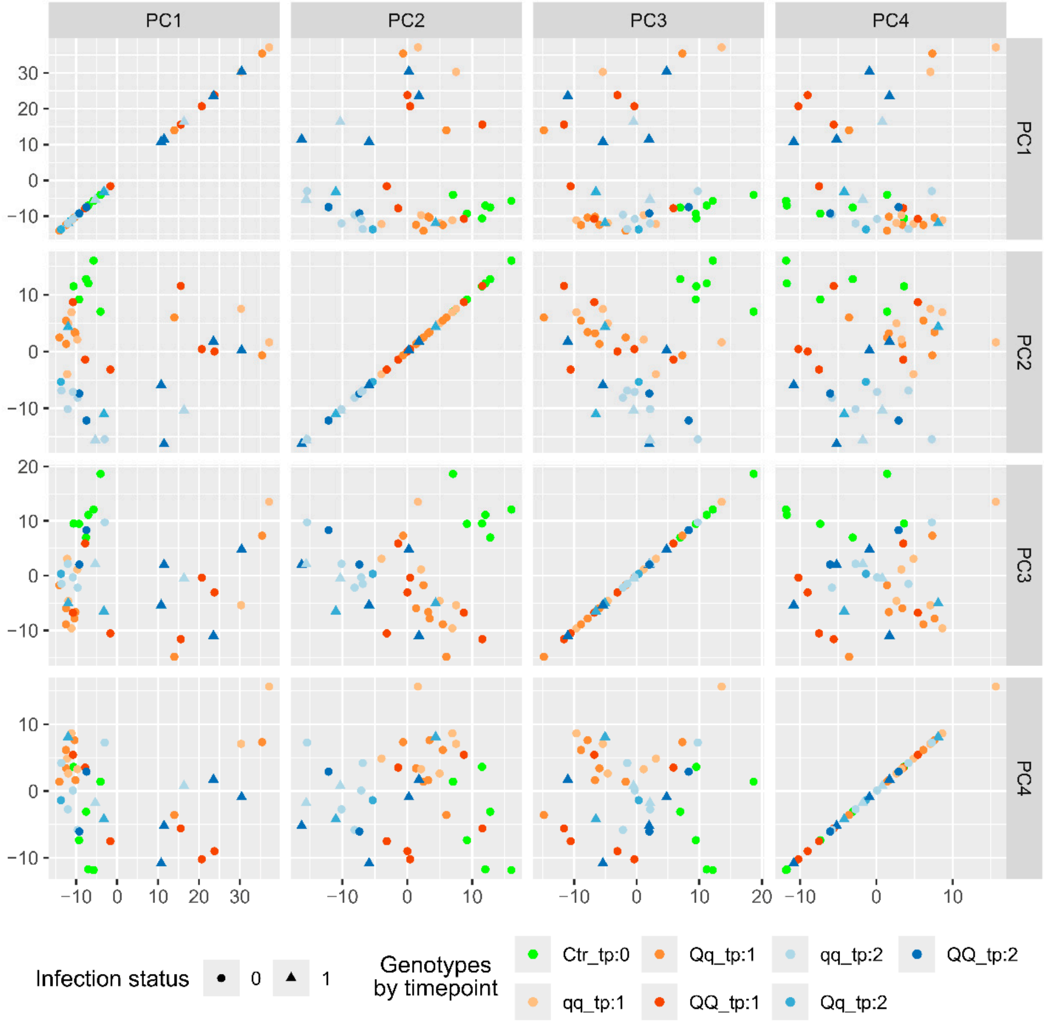

Figure S2. Principal component analysis (PCA) plots from the RNA sequencing analysis from the bucket challenge samples which have been classified by infection status, genotypes and timepoints. Circles (•) correspond to samples with no confirmed qPCR for rIPNV, while triangles (▲) represent qPCR-positive samples. The genotypes representing the IPN-QTL status (QQ, Qq and qq), as well as the sampling timepoints (0, 1 and 2) are colour-coded in the figure.

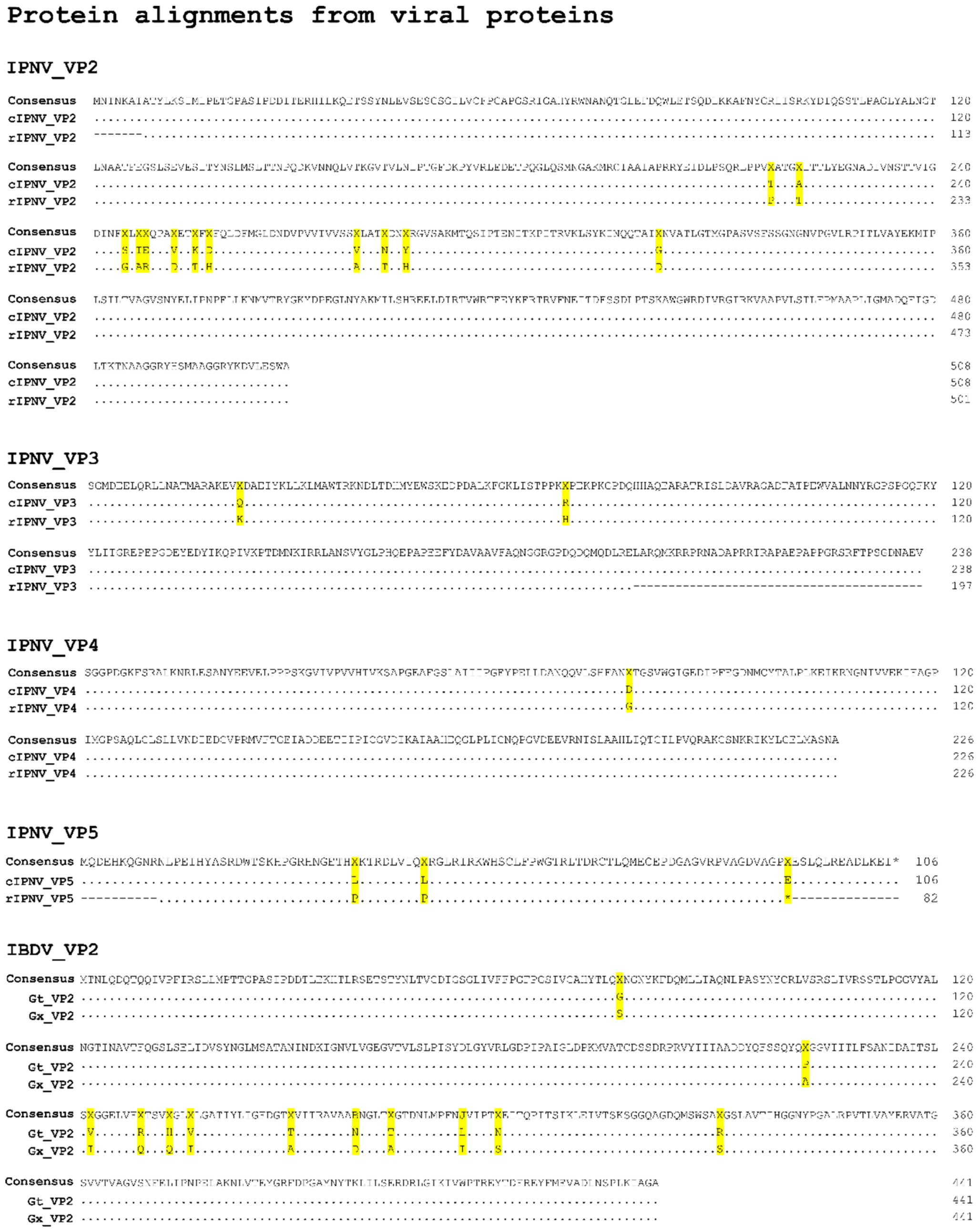

Figure S3. Protein sequence alignments of segment A viral proteins (VP2, VP3, VP4 and VP5) between cIPNV and rIPNV isolates and IBDV-VP2 alignment between Gt and Gx IBDV strains. The consensus protein sequence in one-letter code is shown at the top and the mutations are highlighted in yellow. The dots (.) symbolize conserved residues while the dashes (-) represent missing amino acids in the sequence.

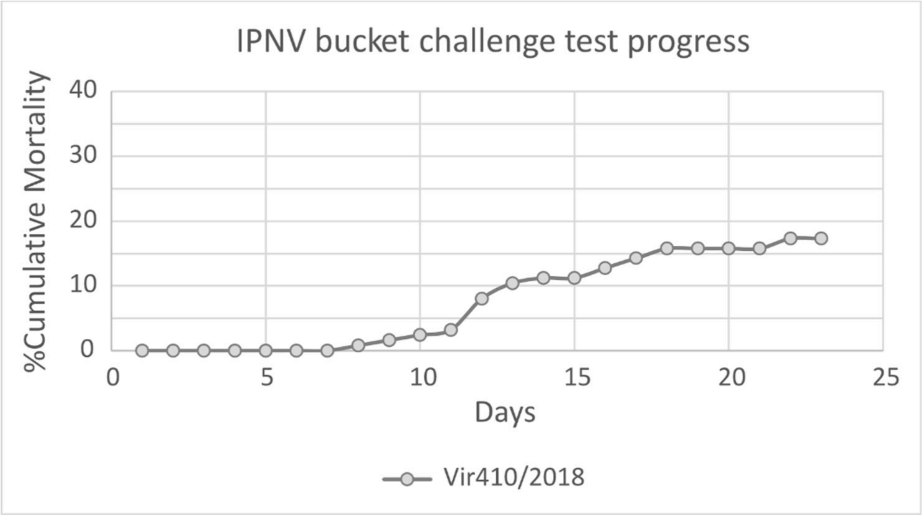

Figure S4. Cumulative mortality during the bucket challenge with rIPNV-infected fish.
